# Prevalence and varieties of complementary and alternative medicine usage among individuals with pre-dialysis chronic kidney disease in Taiwan: an investigative cross-sectional analysis

**DOI:** 10.1186/s12906-023-04311-2

**Published:** 2024-01-02

**Authors:** Ming-Yen Tsai, Yu-Chuen Huang, Ben-Chung Cheng, Chieh-Ying Chin, Yung-Tang Hsu, Wen-Chin Lee

**Affiliations:** 1 Department of Chinese Medicine, Kaohsiung Chang Gung Memorial Hospital and, Chang Gung University College of Medicine, No. 123 Dapi Rd., Niaosong Dist., Kaohsiung, 83301 Taiwan; 2 Department of Medical Research, China Medical University Hospital and School of Chinese Medicine, China Medical University, Taichung, 41354 Taiwan; 3 Division of Nephrology, Department of Internal Medicine, Kaohsiung Chang Gung Memorial Hospital and Chang Gung University College of Medicine, Kaohsiung, 83301 Taiwan; 4 Department of Nursing, MeiHo University, Pingtung, 912009 Taiwan; 5 Kaohsiung Municipal Feng Shan Hospital—Under the management of Chang Gung Medical Foundation, Kaohsiung, 830025 Taiwan

**Keywords:** Complementary therapies, Alternative medicine, Chronic kidney disease, Dietary supplements, Cross-sectional study, Taiwan

## Abstract

**Background:**

Complementary and alternative medicine (CAM) is frequently used in the general population, yet only limited data are available regarding the prevalence of these medications in patients with chronic kidney disease (CKD). Hence, our study aimed to explore the prevalence and types of CAM in Taiwanese patients with CKD.

**Methods:**

A cross-sectional questionnaire survey was conducted by face-to-face interview of 275 pre-dialysis patients without dialysis treatment or kidney transplant at an outpatient nephrology clinic in Taiwan from March 2021 to June 2023. The study outcomes were the prevalence of CAM, CAM types, reasons for using CAM, and sources of information about CAM.

**Results:**

Overall, 128 patients (46.5%) were using CAM, but no significant differences from non-CAM users in the various CKD stages (*p* = 0.156) were found. CAM usage was high in the age range of 20–60 years and duration of CKD ≤ 5 years (*p* < 0.05). The most commonly used type of CAM was nutritional approaches (79.7%), followed by other complementary health approaches (26.6%). The most commonly utilized modalities of CAM were vitamins and minerals (38.3%), and only 27.1% of patients disclosed their CAM use to their physicians. The most common sources of information about CAM were family and friends, cited by 66% of the participants. Health promotion and a proactive attitude were reported by 40% of users as the reasons for using CAM.

**Conclusions:**

The present study provides data on the CAM usage among CKD patients and adds to the increasing evidence on CAM use. Because some of these practices have safety concerns, better education from healthcare providers on the risks and benefits of CAM therapy is needed by CKD patients.

**Supplementary Information:**

The online version contains supplementary material available at 10.1186/s12906-023-04311-2.

## Background

Chronic kidney disease (CKD) is an important and noteworthy disease that is emerging as a major public health issue. The global prevalence of CKD was 10.7% (850 million people) in 2021, when it caused 2.4 million deaths and was one of the top 15 cause of mortality worldwide [1]. CKD places a heavy burden on the medical system because it leads to end-stage renal disease (ESRD), which can significantly reduce quality of life (QOL) and is associated with a high mortality rate. Taiwan has a high prevalence of ESRD, and this situation needs greater attention than it is currently receiving [2].

It is important to ameliorate the deterioration in the glomerular filtration rate (GFR) and educate patients to prevent progression of CKD through multidisciplinary care (MDC) education programs. Once patients make the transition from the pre-dialysis phase to the ESRD phase, they are in an irreversible state and the frequency of adverse events increases [3]. Optimal pre-ESRD nephrology care involves early interventions to modify the risk factors of CKD; the management of comorbidities, malnutrition and metabolic complications; and timely provision of dialysis [4]. Since 2006, the implementation of pay-for-performance (P4P) program initiatives in Taiwan for the education and care of individuals in the pre-dialysis phase has gained recognition as a fitting strategy and a promising approach. This method places a strong emphasis on value-based purchasing, utilizing incentives tied to renal indicators, all with the overarching objective of improving healthcare quality and the long-term outlook for patients in CKD stages 3–5 [5–7]. This collaborative effort of involves working closely with nephrologists, nurses, pharmacists, and dietitians. However, a significant ongoing challenge is the insufficient adherence to these interventions and the recommended lifestyle changes among CKD patients [8].

The non-curative course of the P4P program has pushed patients with CKD to seek alternative therapies to improve their QOL and maintain residual renal function. Complementary and alternative medicine (CAM) is popular with patients with chronic diseases, as previously found in multiple studies worldwide [9, 10]. The average prevalence of CAM users in 32 countries is 26.4%, and it is as high as 50% in China, the Philippines and South Korea [11]. In certain scenarios, CAM has been noted to offer perceived advantages, including the capacity to manage illnesses, address the side effects of conventional medicine, and enhance the QOL and overall well-being, providing a sense of hope for individuals with CKD [12–15]. Nevertheless, a rigorous scientific evaluation of CAM is essential, focusing on its safety, effectiveness, and the quality of CAM treatments, as well as the availability of CAM therapies and their rational utilization [16]. Achieving these goals can be further reinforced through the implementation of suitable public policies, legislation, and the integration of CAM into the national health insurance (NHI) system.

In Taiwan, the NHI program provides full reimbursement for traditional Chinese medicine (TCM) treatments, including Chinese herbal medicine (CHM), acupuncture, and traumatology manipulative therapies, and 45.3% of CKD patients have used TCM, according to the NHI research database [17]. However, the data on the use of CAM among CKD patients remain insufficient. We believe that there are still many instances of CKD patients using other CAM modalities without the knowledge of their medical teams. Thus, we conducted this study to determine the prevalence of CAM usage in pre-dialysis CKD patients in Taiwan. We also explored the types of CAM, demographic factors related to CAM usage, and reasons why pre-dialysis patients use CAM.

## Materials and methods

### Study design and population

This cross-sectional study was conducted at Chang Gung Memorial Hospital (CGMH), a medical center in Kaohsiung, Taiwan, from March 2021 to June 2023. Convenience sampling was adopted in this study to select patients registered in the national pre-ESRD nephrology care program since 2015. Patients older than 20 years, diagnosed as pre-dialysis CKD having eGFR < 45 mL/min/1.73 m^2^ for more than six months were included in the study. Patients were excluded from the study if they were ESRD receiving dialysis treatment, recipients of a kidney transplant, proteinuria without eGFR staging, refused to sign the consent form or were unfit to be interviewed due to cognitive deficits, psychotic disorders, or inability to perform self-care. Face-to-face interviews using a questionnaire were conducted with selected CKD patients at the nephrology clinic after informed consent was obtained.

The sample size was calculated by z test for the difference between two proportions in G power 3.1.9.2. The estimated prevalence of CAM use in CKD patients was taken as 25.2% based on a published study [10]. For a 95% power confidence level and a 5% significant level confidence limit, the minimum sample size required was 275. The study was approved by the Ethics Committee of the KCGMH (No. 202100097B0). This study complies with the STROBE Statement aimed at Strengthening the Reporting of Observational Studies in Epidemiology guidelines and the required information is presented correctly. The STROBE statement checklist for this study is available in Additional file 1.

### Questionnaire

The questionnaire used in the study was adapted from available literature [18–21]. As shown in Additional file 2, it consisted of 28 items on multiple topics, including 9 items on sociodemographic information of the patients and 3 items related to CKD. The remaining 16 questions focused on the details of CAM, such as the types, frequency, reasons, side effects, compliance, and sources of information about CAM use, as well as the disclosure of use and reasons for nondisclosure to physicians. The face validity of the questionnaire was established by 2 physicians, 2 nurses and 1 epidemiologist, all of whom were academicians with research backgrounds in the area of CAM and nephrology. The content validity index (CVI) score was 0.93. A kappa value of 0.84 was obtained from the final version of the questionnaire, indicating excellent inter-rater reliability.

### Definition and classification of CAM

For the purposes of this study, the use of CAM was defined as “any type of CAM used more than twice during the six months after a diagnosis of pre-dialysis.” CAM was classified according to the categories used by the National Center for Complementary and Integrative Health [22]. The three categories are as follows: (1) nutritional approaches such as herbs, vitamins and minerals, and dietary supplements; (2) psychological and physical approaches such as qigong, yoga, acupuncture, exercise, chiropractic, meditation, and tai chi; (3) other complementary health approaches such as Ayurvedic medicine, TCM, homeopathy, and naturopathy. In this study, vitamins and minerals used by patients were not considered CAM if they were prescribed by their physicians. Herbal medicine was defined in this study as an herbal extract purchased by the patients as a decoction, powder or tea from non-medical institutions. Patients were specifically asked if they had used CAM for general health, for the treatment of CKD, or for the treatment of other chronic conditions.

### Study procedure

Patients were contacted by the researchers while they were waiting for their appointments at the nephrology clinic. Participants were briefed on the objectives of the study and the definition of CAM prior to the commencement of the interview. They were shown lists and pictures of different types of CAM prior to the interview in order to assist them to provide accurate information on the type(s) of CAM used. Each interview lasted for 10–15 min.

### Data analysis

Data analysis was conducted using SPSS version 18 (IBM Corp., Armonk, NY, USA). The data were presented in terms of frequencies and percentages. To evaluate the relationship between CAM utilization and sociodemographic variables, as well as its association with various groups of pre-dialysis patients, Pearson's Chi-squared test or Fisher's exact test was employed. A significance level of *p* < 0.05 was applied to all analyses.

## Result

Out of the 300 pre-dialysis patients initially approached for the study, 275 consented to participate, giving a response rate of 91.7%. The majority of the patients were male, were aged more than 60 years, had a low education level, and were married. The sociodemographic details of the study participants are presented in Table 1. Among all the sociodemographic variables, only the age range of 20–60 years old and duration of CKD ≤ 5 years were significantly high in the CAM group (*p* < 0.05).

**Table 1:** Sociodemographic characteristics of CAM and non-CAM users with pre-dialysis

Characteristic	Total (%) *n* = 275	CAM user (%) *n* = 128	Non-CAM user (%) *n* = 147	*p*-value^a^
Gender				0.908
Male	160 (58)	74 (57.8)	86 (58.5)	
Female	115 (41.8)	54 (42.2)	61 (41.5)	
Age (years)				0.020*
20–60	72 (26.2)	42 (32.8)	30 (20.4)	
> 60	203 (73.8)	86 (67.2)	117 (79.6)	
Educational level				0.77
University	26 (9.5)	13 (10.2)	13 (8.8)	
Middle school	142 (51.6)	68 (53.1)	74 (50.4)	
Primary school or below	107 (38.9)	47 (36.7)	60 (40.8)	
Religion				0.681
Buddhism/Taoism	209 (76)	97 (75.8)	112 (76.2)	
Christian/Catholic	12 (4.4)	7 (5.5)	5 (3.4)	
Atheist	54 (19.6)	24 (18.8)	30 (20.4)	
Residence				0.177
Rural	76 (27.6)	30 (23.4)	46 (31.3)	
Urban	199 (72.4)	98 (76.6)	101 (68.7)	
Monthly income (NTD)				0.254
< 40,000	204 (74.2)	89 (69.5)	115 (78.2)	
40,000–50,000	48 (17.5)	26 (20.3)	22 (15)	
> 50,000	23 (8.4)	13 (10.2)	10 (6.8)	
Work status				0.198
Employed	84 (30.5)	44 (34.4)	40 (27.2)	
Unemployed	191 (69.5)	84 (65.6)	107 (72.8)	
Marital status				0.295
Single	27 (9.8)	15 (11.7)	12 (8.2)	
Married	184 (66.9)	88 (68.8)	96 (65.3)	
Divorced/widowed	64 (23.3)	25 (19.5)	39 (26.5)	
Comorbidity				
Hypertension	235 (85.5)	109 (85.2)	126 (85.7)	0.896
Diabetes	126 (45.8)	62 (48.4)	64 (43.5)	0.416
Cardiovascular disease	24 (8.7)	7 (5.5)	17 (11.6)	0.074
Gout	76 (27.6)	29 (22.7)	47 (32)	0.085
Hyperlipidemia	66 (24)	27 (21.1)	39 (26.5)	0.292
Duration of CKD (years)				0.032*
≤ 5	125 (45.5)	67 (52.3)	58 (39.5)	
> 5	150 (54.5)	61 (47.7)	89 (60.5)	

*Abbreviations*: *CAM* complementary and alternative medicine, *CKD* chronic kidney disease

^*^p < 0.05

^a^Chi-squared test

Table 2 presents the patient distribution by severity of CKD. Most of the patients were in Stage 3b of CKD (n = 129, 46.9%), followed by Stage 4 (*n* = 95, 34.5%) and stage 5 (*n* = 51, 18.5%). Among the 275 respondents, 46.5% reported using at least one type of CAM more than three times in the previous 6 months. There were no significant associations between the different groups of patients by CKD severity level and CAM use. In addition, we analyzed the CAM use among different groups of patients with pre-dialysis stratifying by age and duration of CKD as shown in Table 3. There were no significant associations between the different groups of patients by CKD severity level and CAM use stratifying by the age. However, patients with CKD duration ≤ 5 years have significant association between the different groups of patients by CKD severity level and CAM use (*p* = 0.039).

**Table 2:** CAM use among different groups of patients with pre-dialysis

Groups of CKD patients	Total (%) *n* = 275	CAM user (%) *n* = 128	Non-CAM user (%) *n* = 147	*p*-value^a^
Stage 3b	129 (47)	67 (52)	62 (42)	0.156
Stage 4	95 (35)	37 (29)	58 (39)	
Stage 5	51 (19)	24 (19)	27 (18)	

*Abbreviations*: *CAM* complementary and alternative medicine, *CKD* chronic kidney disease

^a^Chi-squared test

**Table 3:** Comparison of age and duration in different CKD stages between CAM users and non-users

Variable	CAM user (%) *n* = 128	Non-CAM user (%) *n* = 147	*p*-value^a^
Age (years) 20–60			0.589
Stage 3b	23 (18.0)	13 (8.8)	
Stage 4	10 (7.8)	10 (6.8)	
Stage 5	9 (7.0)	7 (4.8)	
Age (years) > 60			0.336
Stage 3b	44 (34.4)	49 (33.3)	
Stage 4	27 (21.1)	48 (32.7)	
Stage 5	15 (11.7)	20 (13.6)	
Duration of CKD (years) ≤ 5			0.039*
Stage 3b	33 (25.8)	18 (12.2)	
Stage 4	19 (14.8)	29 (19.7)	
Stage 5	15 (11.7)	11 (7.5)	
Duration of CKD (years) > 5			0.737
Stage 3b	34 (26.6)	44 (30.0)	
Stage 4	18 (14.1)	29 (19.7)	
Stage 5	9 (7.0)	16 (10.9)	

*Abbreviations*: *CAM* complementary and alternative medicine, *CKD* chronic kidney disease

^*^p-value < 0.05

^a^Chi-squared test

As for the types of CAM used by the respondents, this study found that nutritional approaches (*n* = 102, 79.7%) were the most common type of CAM used, followed by other complementary health approaches (*n* = 34, 26.6%). A small portion used mind and body practices (*n* = 14, 11%). In the subgroup analysis, it was found that nutritional approaches were more commonly used by patients in stage 3b (83.6%) and stage 5 (87.5%); however, none of the psychological and physical approaches were reported by patients in stage 5 (Table 4).

**Table 4:** Types of CAM used by different groups of pre-dialysis patients (*n* = 128)

Types of CAM	Total (%)^b^	Groups of CKD patients, n (%)^b^		
		**Stage 3b** ***n* = 67**	**Stage 4** ***n* = 37**	**Stage 5** ***n* = 24**
Nutritional approaches	102 (79.7)	56 (83.6)	25 (67.6)	21 (87.5)
Psychological and physical approaches	14 (11)	10 (14.9)	4 (10.8)	0 (0)
Other complementary health approaches	34 (26.6)	15 (22.4)	14 (37.8)	5 (20.8)

*Abbreviations*: *CAM* complementary and alternative medicine

^b^The total value may be greater than the number of patients, as some could have used more than one type of CAM

Table 5 lists the top 10 CAM modalities used by the various groups of pre-dialysis patients. Vitamins and minerals (38.3%) and CHM (21.9%) were the most common nutritional and other complementary health approaches used, respectively. Among the users of vitamin and mineral users in different stages, those in stage 5 were the largest group (41.7%). However, in comparison with the stage 3b and 4 patients, stage 5 patients were noted to have the lowest usage of other CAM approaches. Interestingly, higher percentages of CAM, including CHM (27%), lutein (27%), acupuncture (16.2%), and exercise (10.8%), were observed in the stage 4 group than in the other patient groups. None reported using herbal medicines or secret recipes.

**Table 5:** Top 10 common CAM modalities used by different groups of pre-dialysis patients (*n* = 128)

Types of CAM	Total (%)^b^	Groups of CKD patients, n (%)^b^		
		**Stage 3b** ***n* = 67**	**Stage 4** ***n* = 37**	**Stage 5** ***n* = 24**
Vitamin & minerals	49 (38.3)	24 (35.8)	15 (40.5)	10 (41.7)
CHM	28 (21.9)	13 (19.4)	10 (27)	5 (20.8)
Lutein	26 (20.3)	12 (17.9)	10 (27)	4 (16.7)
Fish oil	16 (12.5)	11 (16.4)	3 (8.1)	2 (8.3)
Glucosamine	15 (11.7)	10 (14.9)	2 (5.4)	3 (12.5)
Calcium	13 (10.2)	9 (13.4)	2 (5.4)	2 (8.3)
Acupuncture	10 (7.8)	2 (3)	6 (16.2)	2 (8.3)
Probiotics	10 (7.8)	7 (10.4)	2 (5.4)	1 (4.2)
Exercise	10 (7.8)	6 (9)	4 (10.8)	0 (0)
Collagen	4 (3.1)	3 (4.5)	0 (0)	1 (4.2)

*Abbreviations*: *CAM* complementary and alternative medicine, *CHM* Chinese herbal medicine

^b^The total value may be greater than the number of patients, as some could have used more than one type of CAM

**Fig. 1:**
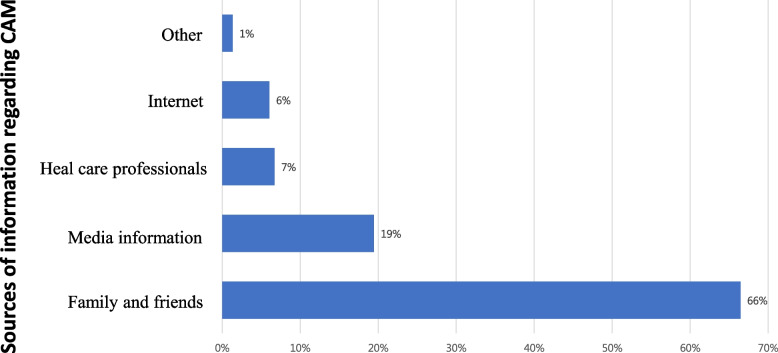
Sources of information regarding CAM. Abbreviation: CAM, complementary and alternative medicine

Two-thirds (66%) of the respondents obtained information about CAM from their family and friends. Some of them depended on information from the internet (6%) and other media sources such television or newspapers (19%). Only 7% of the respondents had ever asked healthcare professionals about CAM (Fig. 1).

**Fig. 2:**
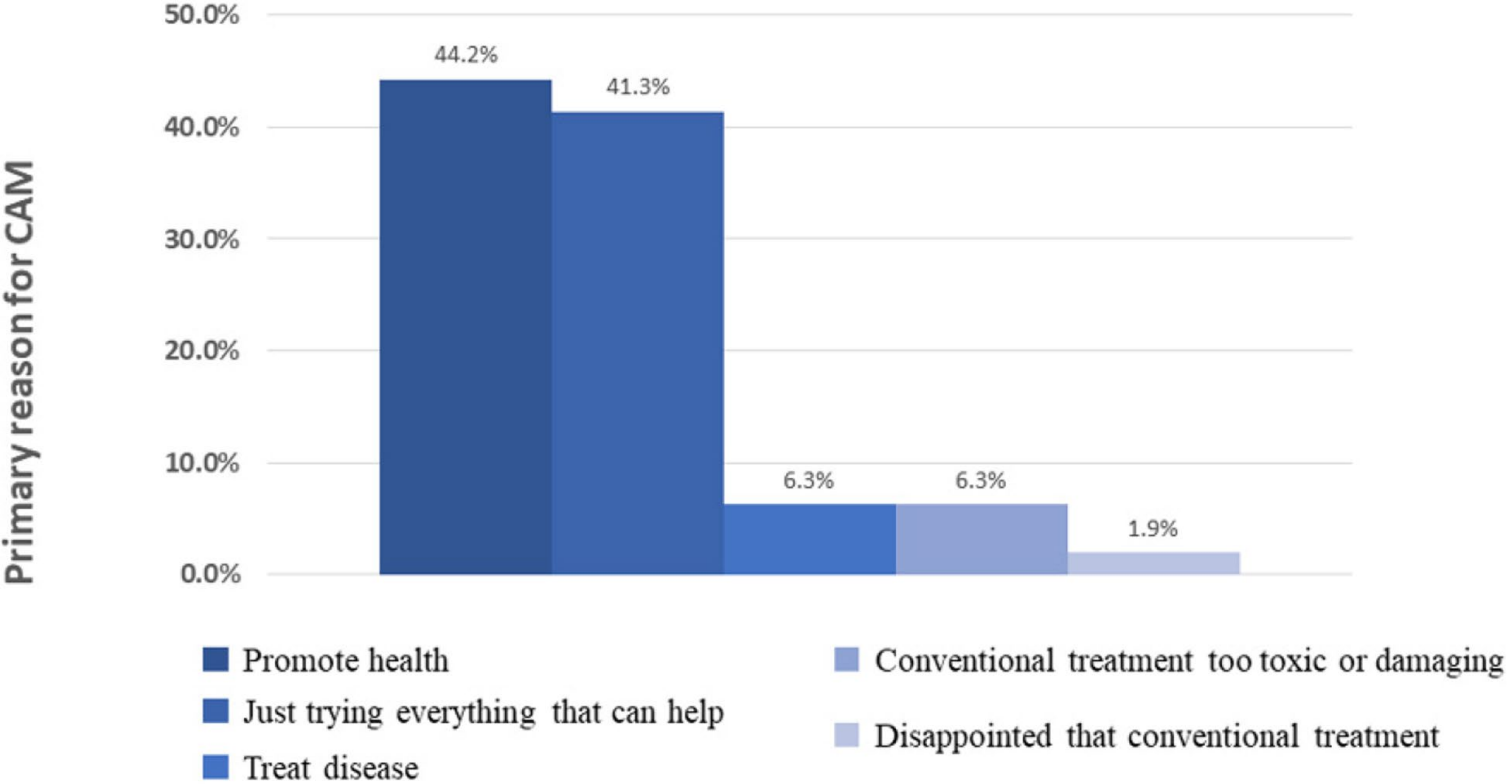
Primary reason for CAM use. Abbreviation: CAM, complementary and alternative medicine

When asked about their purposes in using CAM (Fig. 2), the majority of the patients reported that their usage was driven by a desire for improvement of their general health (*n* = 92, 44.2%), followed by a proactive attitude (*n* = 86, 41.3%). Small minorities used CAM to treat their CKD (6.3%) or considered conventional treatment to be too toxic or damaging (6.3%). Only 4 patients (1.9%) reported using CAM because they were disappointed with conventional treatment.

**Fig. 3:**
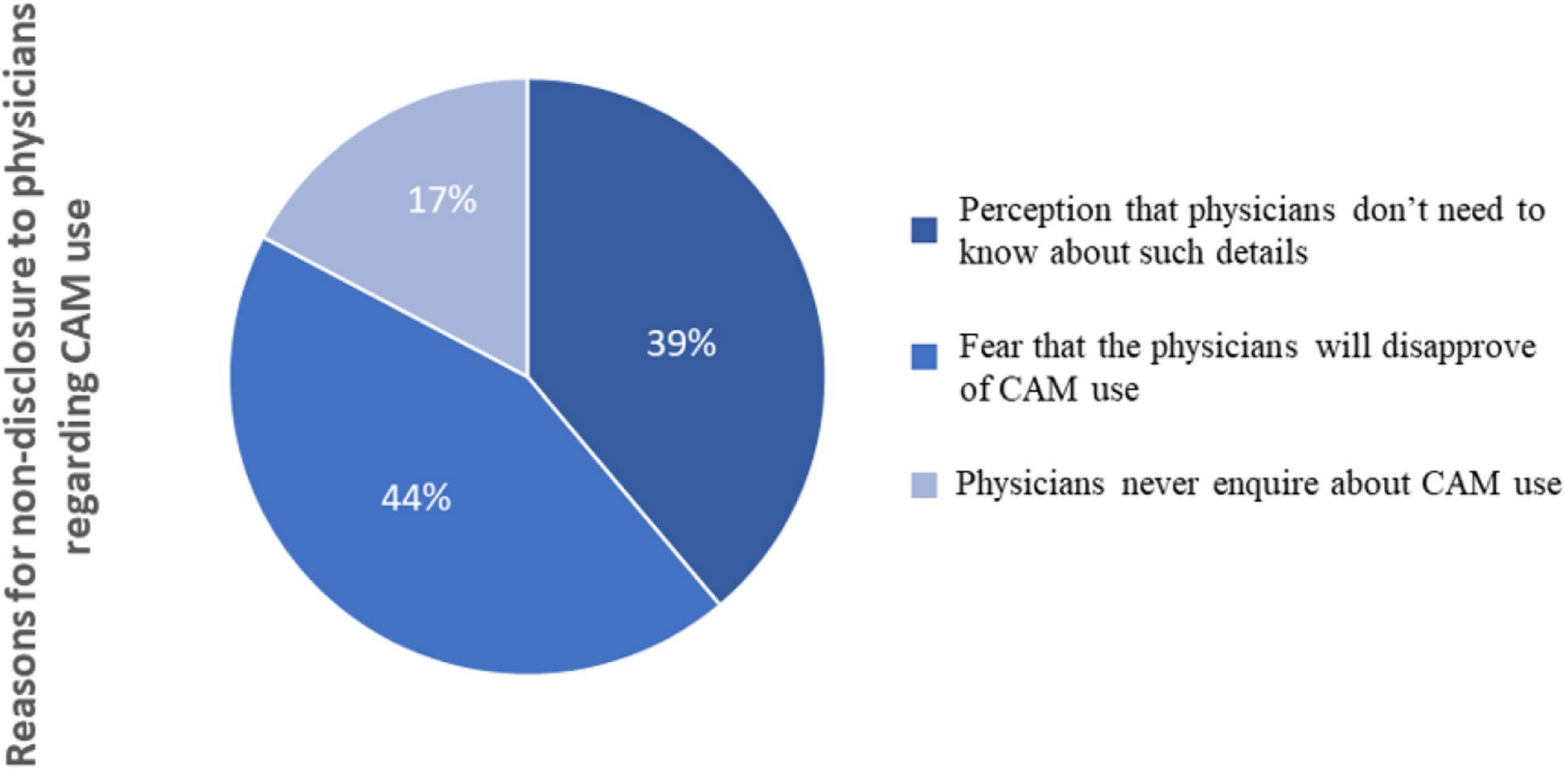
Reasons for non-disclosure of CAM use to physicians. Abbreviation: CAM, complementary and alternative medicine

As high as 72.9% (*n* = 92) of the 125 CAM users among the pre-dialysis patients did not disclose their CAM use to their physicians. Upon further investigation, the most common reason stated for the non-disclosure was that patients feared that the physicians would disapprove of their use of CAM (44%). Another 39% of the patients thought that their physicians did not need to know such details, while 17% of the patients answered that their physicians did not inquire about CAM use (Fig. 3).

More than half (62.5%) of the CAM users expressed satisfaction and 37.5% (*n* = 48) were neutral on the topic of CAM use. No users reported experiencing adverse effects (e.g., vomiting and diarrhea) from CAM use. Compliance with modern medical treatment was affected in 3.9% of the CAM users.

## Discussion

In our sample population, 46.5% of the patients with pre-dialysis CKD reported having used CAM within half a year of the survey. The most common modality used by patients was nutritional approaches. A majority of patients felt that the CAM therapy was very important for their general health. An estimated two-thirds of the patients obtained CAM information from their family and friends, and 72.9% of them did not disclose their CAM use to their physicians.

In comparison to other international surveys among similar CKD patients, the prevalence of CAM use was lower in our study than in Egypt (64%) [23] but higher than in Turkey (25.2%) [9], Malaysia (29%) [24] and Korea (24.6%) [25]. The variation in findings can be attributed to disparities in how CAM usage is defined, the diverse geographical locations, cultural influences, and socioeconomic statuses considered in these studies. Additionally, the differing official policy stances and empirical support for CAM use within the CKD population could play a role in this discrepancy. While most of the research conducted to identify patterns of CAM usage have focused only on the general CKD population [9, 23–25], limited information on the prevalence of CAM usage by pre-dialysis patients utilizing preventive services has been reported. To the best of our knowledge, this is the first study to be reported from Taiwan.

Upon commencement of the P4P program, the care indicators include renal function maintenance, improvement of proteinuria, continuous MDC, pre-inserted hemodialysis (HD) access, and pre-ESRD management and education [26]. Although the P4P program can significantly mitigate the HD events, hospitalization, and all-cause mortality in patients with CKD stages 3–5 compared to patients who do not receive it [7], such medical care is not completely achievable. A recent study found that only 37% of HD institutes that intensively performed pre-ESRD nephrology care optimally responded to anemia and prepared an arteriovenous fistula for HD [27]. The keys to success have been reported as a more positive attitude toward the disease, a willingness to cooperate with treatment adherence, and self-management, such as the adoption of nutritional counseling, lifestyle behavior changes, and avoidance of NSAIDS, nephrotoxins and unproven therapies [28]. Even under the comprehensive pre-ESRD nephrology care, the additional choice of CAM use is common.

In our study, the prevalence of CAM usage was high in the group aged 20–60 years, according to the distribution. This finding was consistent with a study by Birdee et al*.* [29] and Castelino et al*.* [30]*,* which showed that CAM usage was higher in young and middle-aged patients than in other age groups. The prevalence of CKD is higher in this age group due to a higher incidence of chronic illness such as diabetes and hypertension [31]. Meanwhile, the use of CAM also varies by duration of CKD. Our study found that the prevalence of CAM usage in patients with stages 3b, 4, and 5 show no significant differences compared with subgroups of non-CAM users. While most of the studies conducted by the national surveys of some countries to report the high prevalence of CAM usage have been at advanced stages of CKD [24, 30, 32], very few surveys have reported CAM prevalence among patients with middle stage. To address this gap, we further analyzed the usage of CAM according to different stage groups stratifying by age and duration of CKD. Our findings revealed a significant influence between CAM usage and patients diagnosed within 5 years of CKD 3b, whereas age was not relevant. This situation may be explained by participants who are diagnosed with CKD 3b in the P4P program more willing to spend on CAM methods in conjunction with their conventional therapies. Notably, the current clinical practice guidelines primarily focus on ambulatory care, including monitoring underlying conditions and implementing diet and lifestyle controls [33, 34]. At a more advanced stage, the intervention of conventional medications increases, and the illness lasts longer, there are signs of a decline in CAM treatments.

The prevalence found in our study was relatively low compared to the CAM usage in the general population (75.5–86.9%) in Taiwan [35–37] and in patients with other chronic illnesses [38, 39]. This difference may be due to increased public awareness of the risk of kidney problems with CAM use following media campaigns. Meanwhile, the use of CAM to treat kidney disease was found to be low in our study (6.3%), as also found in another study in Turkey [9]. The majority of the patients used CAM to promote their health (44.2%) and maintain a proactive attitude (41.3%). This finding may be partially related to the early patient education, in which patients with pre-dialysis learn that disease progression is irreversible. It also confirms that patients do not use CAM to replace conventional medicine but rather to complement it. However, the diverse cultural beliefs in Taiwan still lead to misconduct in looking for health behaviors, such as purchasing CAM products and following guidance from non-medical sources [18]. Our study found that over 70% of the study participants learned about CAM therapies from family/friends and about 25% from the media. The main reason why patients did not inform their physicians about their CAM use was concerns about physicians' disapproval of the use of CAM, which reflects poor patient–physician communication. Many of the CAM users kept their use of CAM from their physicians, as reported in the present study and other similar studies [23, 40, 41], simply because their healthcare providers did not inquire about it. Therefore, healthcare providers must be aware of CAM usage in their patients and inquire about such practices, as CAM may interact with prescribed medications and affect patient compliance with the care plan [42, 43].

In this study, 79.7% of the CAM users reported having used nutritional supplements, and most of these frequently relied on vitamins and minerals, lutein, fish oil, glucosamine, calcium, etc. Patients with CKD may experience vitamin deficiency, hypertension, hyperphosphatemia, anorexia, as well as protein-energy wasting. Medical nutritional therapy is recommended to delay CKD progression and to prevent and/or treat malnutrition and wasting [44]. Otherwise, our results should serve as a reminder to healthcare professionals that the proportion of patients purchasing over-the-counter medications cannot be ignored, and clinicians must be aware of serious possible sequelae. For example, multivitamins are not recommended in CKD stages 3–5 because many provide higher dosages of vitamins A, E, and K, which can lead to anemia, liver dysfunction, heart failure, or coagulopathy [45, 46]. Excessive vitamin C intake can increase oxalate excretion, increasing the risk of kidney stones and body tissue deposition [47]. Minerals such as potassium, calcium, magnesium and phosphorus also have the potential to worsen kidney function [48]. Even though fish oil has been proven to improve blood pressure, lower blood triglycerides, reduce cardiovascular events, and at the same time reduce the inflammatory response of the kidneys and protect them [49], excessive use still entails a risk of bleeding [50]. Although most dietary supplements are known to have antioxidant effects which, through multiple mechanisms, can prevent aggravated glomerulosclerosis and fibrosis during CKD [51], there is little evidence that they improve CKD outcomes [52].

CHM and acupuncture use were reported by 21.9% and 7.8% of the CAM users, respectively. These rates of TCM consumption are higher than those reported in an earlier survey of CKD patients in northern Taiwan [53]. In a chart review survey of 8459 pre-dialysis patients [53], only 4.8% were currently using TCM and preferred CHM over other TCM services. Although certain CHM therapies analyzed in population-based studies have scientific merit and evidence supporting their use [17], some traditional herbs lack such support and might be potentially harmful to patients [54, 55]. Unexpectedly, the use of herbs/recipes was reported by 0% of the CAM users in the present study. This result shows that our pre-dialysis patients were less likely to seek non-prescribed CHM from herbal and supplement stores. Since 1995, the CHM prescribed by TCM practitioners under the NHI program has been mostly a scientifically-designed powder, which is convenient and well preserved, has a standardized GMP manufacturing process, and meets standards for heavy metals, flatoxin, and pesticide residues [56]. The prescribed CHM is known to reduce the mortality rate in patients with CKD by 40% as compared with non-users [57]. Other studies also reflect that CKD patients who receive proper CHM prescriptions might obtain more beneficial effects, including maintained eGFR in the advanced stage [58], improved QOL [53], and prolonged time to dialysis in patients with diabetes [59]. Patients with CKD and several comorbidities may simultaneously take many different medications. Therefore, the additional intake of either nutritional products or CHM for CKD is usually complicated and needs to be integrated due to the increased risk of side effects from drug–nutrient or drug–herb interactions.

Our study offers valuable insights to healthcare providers regarding the utilization of CAM among pre-dialysis CKD patients. It is imperative to conduct comprehensive research to understand the frequency and interactions of CAM therapies with prescribed medications in CKD patients. To achieve this, the MDC team should comprise experts well-versed in CAM, capable of supplying relevant evidence regarding its advantages and potential risks. Moreover, the persistent use of CAM in current populations is a critical aspect to monitor in relation to the progression of future diseases.

This study has several limitations. First, as it was conducted in southern Taiwan at a hospital with an outpatient nephrology clinic and the sample was recruited by the convenience sampling method, the results cannot be generalized to other populations. Second, it is possible that the level of use of CAM reported was lower than the actual level, as these P4P participants might have been educated to be cautious about CAM usage. Third, there was also the possibility of recall bias regarding the use of CAM in this cross-sectional design. Besides, the Neyman bias is another possible limitation, because we could not identify whether the participants had used CAM in the past or only in the present. Therefore, the results of this study may be affected by prevalence–incidence (Neyman’s) bias. Further prospective cohort studies, which select the newly identified cases as study participants, will effectively avoid Neyman bias.

## Conclusion

CAM use, in particular nutritional approaches, is common in patients with pre-dialysis on the P4P program in Taiwan and is used by a greater percentage of non-elderly group and shorter duration of CKD. Some of the CAM used by patients can potentially lead to adverse side effects or produce unintended interactions with conventional medications. The majority of the patients did not disclose their use of CAM to their physicians because they were worried that the physician would prohibit their use. Thus, it is critical for healthcare professionals to integrate information from patients’ CAM usage and educate them according to evidence-based practice. More studies concerning the safety and efficacy of CAM in the management of CKD in the P4P program should be conducted to achieve better healthcare quality and disease outcomes if any benefits are supported.

### Supplementary Information

**Additional file 1.** STROBE Statement—Checklist of items that should be included in reports of *cross-sectional studies.*

**Additional file 2. **Complementary and alternative medicine questionnaire.

## Data Availability

The datasets used or analyzed (or both) in the study are available from the corresponding author upon reasonable request.

## Abbreviations

CAM: Complementary and alternative medicine
CHM: Chinese herbal medicine
CKD: Chronic kidney disease
CVI: Content validity index
ESRD: End-stage renal disease
GFR: Glomerular filtration rate
HD: Hemodialysis
MDC: Multidisciplinary care
NHI: National Health Insurance
P4P: Pay for performance
QOL: Quality of life
TCM: Traditional Chinese medicine
