# Supplementary material for: Prevalence and varieties of complementary and alternative medicine usage among individuals with pre-dialysis chronic kidney disease in Taiwan: an investigative cross-sectional analysis
Source: BMC Complement Med Ther. 2024 Jan 2;24:11. doi: 10.1186/s12906-023-04311-2 (PMC10759758; doi:10.1186/s12906-023-04311-2)
Supplement: Supplementary file 2 — Additional file 2. Complementary and alternative medicine questionnaire. [file 12906_2023_4311_MOESM2_ESM.docx]

| **Measures** | **Q part** | **Q No** | **Question** | **Response options** | **Additional free text option** |
| --- | --- | --- | --- | --- | --- |
| Patient characteristic | Part 1 | 1 | Age | Open |  |
|  | Part 1 | 2 | Gender | Male/Female |  |
|  | Part 1 | 3 | Educational level | Multiple choice |  |
|  | Part 1 | 4 | Work status | Multiple choice |  |
|  | Part 1 | 5 | Monthly income | Multiple choice |  |
|  | Part 1 | 6 | Residence place | Multiple choice |  |
|  | Part 1 | 7 | Religion | Multiple choice^c^ | Yes |
|  | Part 1 | 8 | Marital status | Multiple choice |  |
|  | Part 1 | 9 | Comorbidity | Multiple choice^c^ | Yes |
|  | Part 1 | 10 | Duration of CKD | Open |  |
|  | Part 1 | 11 | CKD stage | Multiple choice |  |
|  | Part 1 | 12 | Causes of CKD | Multiple choice^c^ | Yes |
| CAM use after CKD diagnosis | Part 2 | 1 | Whether use CAM before CKD diagnosis? | Yes/No |  |
|  | Part 2 | 2 | Whether use CAM in recent 6 month? | Yes/No |  |
|  | Part 2 | A1 | Use of specific CAM therapy and the frequency^a^ | Multiple choice^c^ | Yes |
|  | Part 2 | A5 | What’s the initial symptoms? | Multiple choice | Yes |
|  | Part 2 | A6 | The duration of using CAM | Multiple choice |  |
| Source of information, costs | Part 2 | A4 | Source of information about CAM | Multiple choice^c^ | Yes |
|  | Part 2 | A2 | Average CAM cost (NTD/month) | Multiple choice |  |
| Reasons | Part 2 | A3 | Reasons for CAM use | Multiple choice^c^ | Yes |
|  | Part 2 | B1 | Reasons for not using CAM | Multiple choice^c^ | Yes |
| Adverse effect | Part 2 | A11 | Adverse effect | Yes/No | Yes^b^ |
| Satisfaction | Part 2 | A12 | Satisfaction with CAM | Multiple choice |  |
|  | Part 2 | A13 | Will you continue to use CAM? | Yes/No |  |
| Dialogue about CAM | Part 2 | A7 | Informed doctor about CAM use | Yes/No |  |
|  | Part 2 | A8 | Reasons for not telling doctor | Multiple choice^c^ | Yes |
|  | Part 2 | A9 | Doctor inquired about CAM | Yes/No |  |
| Drug compliance | Part 2 | A10 | CAM use and adherence with treatment | Yes/No |  |

Abbreviation: CAM, complementary and alternative medicine; Q part, question part; Q No, question number; NTD, New Taiwan dollar

^a^ List of 27 specific methods, space for additional therapies and specification. e.g. on type of dietary supplement or on folk medicine, on type of exercise, religious ceremony, modalities of other complementary health approaches and space for other and unknown therapies. Also there are multiple choice for frequency and whether use before CKD diagnosis.

^b^ Free text option if had adverse effects after using CAM

^c^ A multiple answer question
